# A flow cytometry technique to study intracellular signals NF-κB and STAT3 in peripheral blood mononuclear cells

**DOI:** 10.1186/1471-2199-8-64

**Published:** 2007-07-31

**Authors:** Sandrine Lafarge, Hind Hamzeh-Cognasse, Patricia Chavarin, Christian Genin, Olivier Garraud, Fabrice Cognasse

**Affiliations:** 1 EFS Auvergne-Loire, Saint-Etienne, France; 2 GIMAP EA3064, Faculty of Medecine, University of Saint-Etienne, France

## Abstract

**Background:**

Cytokines have essential roles on intercellular communications and are effective in using a variety of intracellular pathways. Among this multitude of signalling pathways, the NF-κB (nuclear factor kappaB) and STAT (signal transducer and activator of transcription) families are among the most frequently investigated because of their importance. Indeed, they have important role in innate and adaptive immunity. Current techniques to study NF-κB and STAT rely on specific ELISAs, Western Blots and – most recently described – flow cytometry; so far, investigation of such signalling pathways are most commonly performed on homogeneous cells after purification.

**Results:**

The present investigation aimed at developing a flow cytometry technique to study transcription factors in various cellular types such as mixtures of B-cells, T-lymphocytes and monocytes/macrophages stimulated in steady state conditions (in other words, as peripheral blood mononuclear cells). To achieve this goal, a two step procedure was carried out; the first one consisted of stimulating PBMCs with IL1β, sCD40L and/or IL10 in such a manner that optimal stimulus was found for each cell subset (and subsequent signal transduction, therefore screened by specific ELISA); the second step consisted of assessing confirmation and fine delineation of technical conditions by specific Western-Blotting for either NF-κB or STAT products. We then went on to sensitize the detection technique for mixed cells using 4 color flow cytometry.

**Conclusion:**

In response to IL1β, or IL10, the levels of phosphorylated NF-κB and STAT3 – respectively – increased significantly for all the studied cell types. In contrast, B-cells and monocytes/macrophages – but, interestingly, not T-lymphocytes (in the context of PBMCs) – responded significantly to sCD40L by increasing phosphorylated NF-κB.

## Background

Cytokines have essential roles in positive, negative and regulatory control of immune responses in every compartment. The biological functions of cytokines mainly depend on cytokine-mediated gene activation or repression. Studies on gene induction by different cytokines led to the discovery of several signalling pathways including NF-κB, STAT and others [1,2].

Various types of specific or innate stimuli activate and phosphorylate the latent cytoplasmic NF-κB/IκB complex, which is ensued by the proteolysis of IκB. The NF-κB p65 sub-unit is then released; it is translocated to the nucleus and binds the genes containing κB sites which in turn, upregulates the expression of these gene products [2-6]. A large variety of molecules are able to activate NF-κB such as PAMPs, IL1, TNF, CD40L, BAFF [2,5]. Among activated transcription factors, the NF-κB family is essential for inflammation, innate and specific immunity, cell proliferation and apoptosis. NF-κB is involved in pro-inflammatory cytokine secretion, anti-apoptotic gene expression and cell proliferation, therefore, altered NF-κB expression or regulation is involved in immune defaults resulting in cancer [7-10] and chronic inflammatory diseases [11].

For the STAT signalling pathway, the process is slightly different than for NF-κB. The binding of certain cytokines (such as IFN, some gp130, IL10, IL3 family, single chain family, IL4...) [1,12] to cell-surface receptors results in receptor dimerization and subsequent activation of JAK tyrosine kinases, which are constitutively associated with these receptors. Tyrosine residues on the receptor (intracellular) tails are then phosphorylated by activated JAKs and serve as docking sites for a family of latent cytoplasmic transcription factors known as STATs. Members of the STAT family are phophorylated by different JAKs (JAK1, JAK2, JAK3 and TYK2) [1]; then, the relevant STAT dimerizes, subsequently leaves the receptor and translocates to the nucleus, where it activates the corresponding gene transcription. There are to date seven human STATs: STAT 1, STAT2, STAT3, STAT4, STAT5A, STAT5B and STAT6 [1,12-14].

Various solid tumors – as well as malignant hemopathies (lymphomas and leukaemias) – in humans have been shown to possess aberrant STAT activation. This property is also found – and can be explored – in cell lines derived from solid tumours or leukemias. For example, among the STAT family, STAT3 is frequently activated in both multiple myeloma cell lines [15,16] and tumors derived from patient bone marrows [17]. STAT3 activation is required for promotion of tumor cell survival and directly contributes to the malignant progression of multiple myeloma by allowing accumulation of long-lived plasma cells [18-20].

The present investigation will focus on the two principal intracellular pathways translocating into the nucleus – for the first – the NF-κB activation pathway (mediated by the integration of a cytokine-derived signal such as by IL-1β or by sCD40L), and – for the other – JAK following activation of the STAT3 pathway (mediated by the integration of a cytokine-derived signal such as by IL10). Those signalling pathways represent the majority of cytokine-delivered stimuli on receptors displayed on the surface of hematopoietic cells and, by extention, of immune cells [21,22]. Currently, studying NF-κB and STAT3 is possible in ELISA [23], Western Blot [24-26] and flow cytometry with purified cells [4]. The first disadvantage of these three techniques is the pretreatment before analyzing: nuclear isolation which takes a long time and requires an important vigilance. The second disadvantage is the under-estimation of the relationship between the different cellular types. We thus aimed to examine nuclear factor transcription during in vitro activation; this flow cytometry technique is convenient in most laboratories. This gave several advantages (feasibility, sensitivity, reproducibility and ease) over other measurements of nuclear factors (confocal microscopy or EMSA). Thus, we developed a flow cytometry technique that studies transcription factors of different cellular types (B-cells, T-lymphocytes and monocytes/macrophages) in homeostasis conditions, in other words without preliminary cell purification. To validate this technique, we compared our data with other techniques.

We present here an original, convenient, and reproducible technique to study various cell (B-cells, T-lymphocytes and monocytes/macrophages) activation events (depending on membrane markers) through nuclear factor translocation (NF-κB and STAT3) by means of a novel, specific flow cytometry assay. The stimuli chosen were IL1β and sCD40L to activate the NF-κB pathway and IL10 to stimulate the STAT3 pathway. Furthermore, these cytokines play a major role in immune responses. The originality of this new technique consists in studying non-purified cells, like some other studies [4,27] but various cells (such as PBMCs) in a likely physiological context.

## Results

### Optimization of culture conditions and stimulation for studying transcription factors

As described, screening procedures to analyse phosphorylated products of the NF-κB or STAT3 pathways prior to flow cytometry, were performed using ELISA and Western Blot techniques. The aim of those preliminary experiments was double: -i) to set up optimized conditions of cell stimulation; -ii) to obtain-conventional – baseline data to be compared with results obtained in the novel – target – technique using detection by flow cytometry.

**Figure 1:**
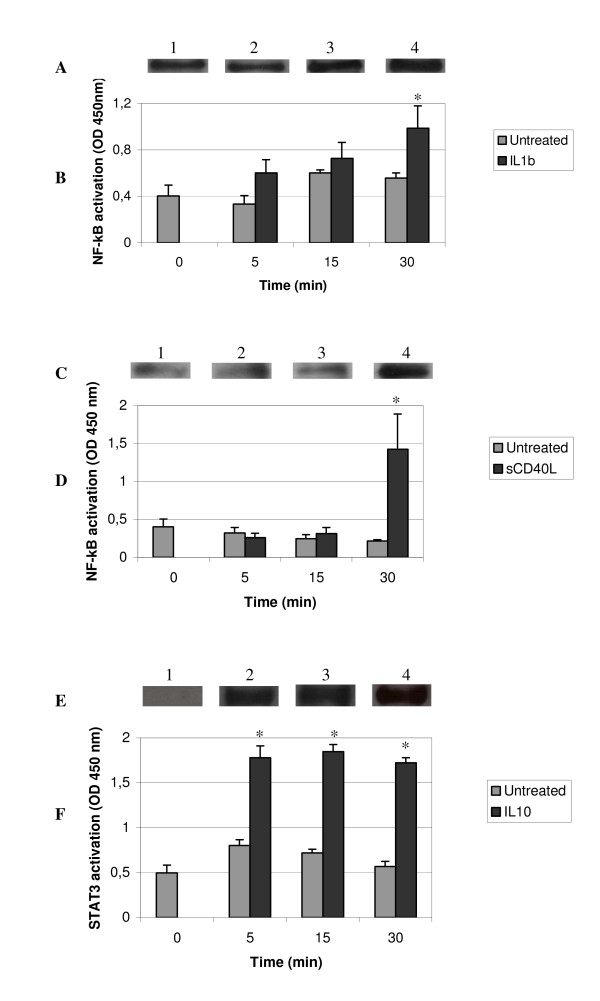
**Western blot and ELISA analysis of phosphorylated NF-κB and STAT3 in PBMCs**. Western Blot of pNF-κB (A, C) and pSTAT3 (E) in PBMCs nuclear extract was observed with or without stimuli: IL1β (50 ng/mL) (A), sCD40L (50 ng/mL) (C) or IL10 (100 ng/mL) (E) for various periods (0 to 30 min). Data were representative of five experiments. pNF-κB and pSTAT3 were detected by ELISA (n = 5) in activated PBMCs nuclear extracts and the time dependant effect of the PBMCs stimulus was observed. PBMCs were stimulated for various periods of time (0 to 30 min) with or without IL1β (B), sCD40L (D) or IL10 (F). Statistical significance (wilcoxon paired test; p < 0.05) was represented by an asterisk (*). Data represented the mean (± SD) of five experiments.

Firstly, PBMCs were stimulated by various activators (i.e. cytokines) at different times (Figure 1). To determine the optimal concentration of each stimulus, we stimulated PBMCs with each activator at various concentrations (IL1β: 0 to 1000 ng/mL; sCD40L: 0 to 1000 ng/mL or IL10: 0 to 200 ng/mL. After PBMC stimulation in 200 μL complete medium for 48 hours at 37°C with 5% CO_2_, individual culture supernatants were removed, filtered (0.22 μm), and frozen until use. After, the cytokine IL-6 (PBMCs activation marker) was measured in individual culture supernatants using commercial specific ELISA kits (R&D Systems Europe Ltd) according to the manufacturer's instructions. Optimal concentration of PBMCs stimulation was obtained with the following concentration, 50 ng/mL of IL1β, 50 ng/mL of sCD40L and 100 ng/mL of IL10. The optimal concentration for each activator conformed to standards in our laboratory [4,28]. In unseparated cell culture conditions (i.e. PBMCs) we could observe – by either ELISA or WB technique – that intracellular pNF-κB concentration was increased following PBMC stimulation with IL1β (Figure 1A, B) and with sCD40L (Figure 1C, D). Maximum phosphorylation and subsequent product detection was obtained for a 30 min stimulation with 50 ng/mL of IL1β and sCD40L (p < 0.05) for 10^6 ^cells (respectively, 0.988 ± 0.190 and 1.423 ± 0.464) versus unstimulated cells (respectively, 0.557 ± 0.044 and 0.212 ± 0.020). These conditions were considered to represent the baseline working culture conditions from which to observe any significant difference of pNF-κB concentration between non-activated PBMCs and PBMCs activated by IL1β or sCD40L.

A similar screening protocol was set up to standardize an optimal pSTAT3 PBMCs response following IL10 treatment of PBMCs, using Western Blot and ELISA. We indeed show (Figure 1E, F) that pSTAT3 concentration is amplified with IL10 stimulation: maximal signal product is obtained after a 5 min exposure of PBMCs to IL10 stimulation used at 100 ng/mL (1.724 ± 0.056) versus unstimulated cells (0.568 ± 0.055). The same signal intensity is achieved for all of the time exposures tested.

### Development of an original flow cytometry technique to study nuclear transcription factors

Having identified optimal conditions for stimulating unseparated PBMCs for the activation of the nuclear translocation factors pNF-κB and pSTAT3, we set up a flow cytometry technique allowing the study of these nuclear factors at the level of PBMC cellular subpopulations without being obliged to purify these cellular subpopulations in e.g. T-lymphocytes, B-cells, monocytes/macrophages, as was previously a prerequisite.

**Figure 2:**
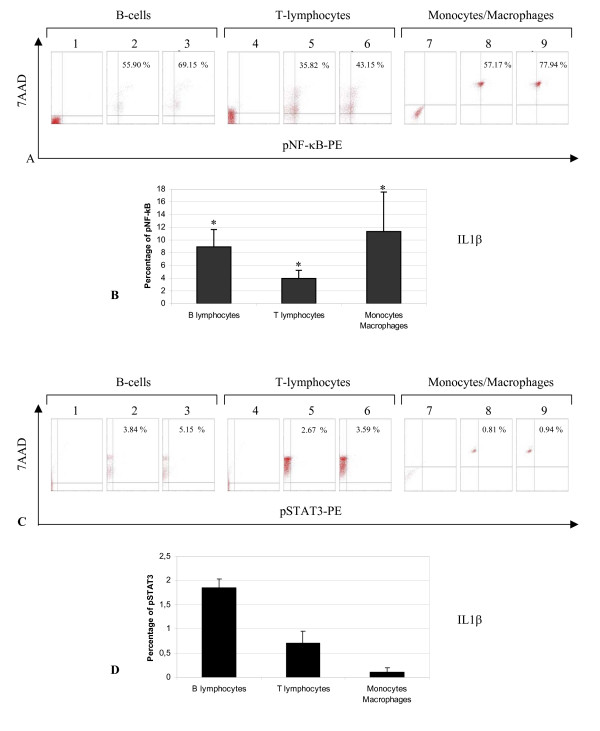
**Flow cytometry of simultaneous phosphorylated NF-κB and STAT3 expression from B-cells, T-lymphocytes and monocytes/macrophages stimulated by IL1β (control)**. Cytogram (A and C) depicted one experiment and showed the isotype control (A1 and C1 for B-cells, A4 and C4 for T-lymphocytes and A7 and C7 for monocytes/macrophages). Cytogram (A) showed the pNF-κB translocation of B-cells (A2, A3), T-lymphocytes (A5, A6) and monocytes/macrophages (A8, A9) with (A3, A6, A9) or without (A2, A5, A8) stimulus IL1β (50 ng/mL) for 30 min. Cytogram (C) showed the pSTAT3 translocation of B-cells (C2, C3), T-lymphocytes (C5, C6) and monocytes/macrophages (C8, C9) with (C3, C6, C9) or without (C2, C5, C8) stimulus IL1β (50 ng/mL) for 30 min. Data were representative of seven experiments. Summary of flow cytometry analysis (n = 7) of percentage of phosphorylated NF-κB (B) and phosphorylated STAT3 (D) activation (versus untreated) from B-cells, T-lymphocytes and monocytes/macrophages. The graphs represent the difference in percentage of phosphorylated nuclear factor between stimulated and untreated cells. Statistical significance (wilcoxon paired test; p < 0.05) was represented by an asterisk (*). Data represented the mean (± SD) of seven experiments.

We show an example of a cytogram that can be obtained with this novel technique to detect transcription factors by means of flow cytometry (Figure 2). The background signals were determined using relevant isotype control antibodies (Figure 2A1, A4, A7 and 2C1, C4, C7). 7AAD negative/pNFkB positive targets are cellular debris – not e.g cytoplasmic NFkB signal. The blots were not always clearly separated and individualized every time. As each cell was not activated to the same extent or level, we were able to observe the activation level from partially activated cells, and from transiently activated cells or non activated cells [29]. As can be seen in Figure 2A and 2C, within different unseparated PBMC subpopulations, CD19^+ ^B-cells (Figure 2A2, A3 and 2C2, C3), CD3^+ ^T-lymphocytes (Figure 2A5, A6 and 2C5, C6), and CD14^+ ^monocytes/macrophages (Figure 2A8, A9 and 2C8, C9) were cultured in the presence (Figure 2A3, A6, A9 and 2C3, C6, C9) or absence (Figure 2A2, A5, A8 and 2C2, C5, C8), of optimal concentrations of IL1β (50 ng/mL) for 30 min at 37°C (as described previously). IL1β induced respectively an increase in nuclear pNF-κB translocation by a magnitude (vs baseline level), of 8.92 ± 2.73% for B-cells, of 3.93 ± 1.31% for T-lymphocytes and of 11.31 ± 6.22% for monocytes/macrophages. However, IL1β did not induce any significant increase of phosphorylated STAT3: 1.85 ± 0.18% for B-cells, 0.69 ± 0.25 for T-lymphocytes and 0.10 ± 0.09 for monocytes/macrophages. The shown cytogram is representative of seven independent experiments. The integration of the sum of individual results is represented in the histograms shown in Figure 2B (NF-κB) and 2D (STAT3): for all the diverse unseparated subpopulations, the level of nuclear pNF-κB translocation increased significantly (p < 0.05) in response of IL1β under defined conditions (this concerns equally B-cells, T-lymphocytes and monocytes/macrophages) contrary to the level of nuclear pSTAT3 translocation.

**Figure 3:**
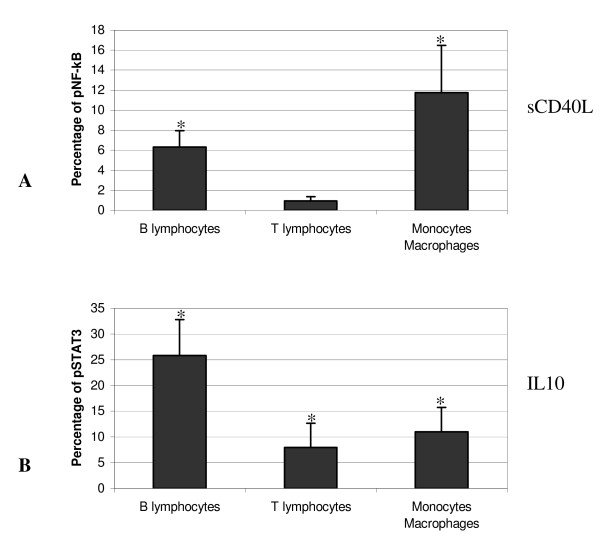
**Flow cytometry of simultaneous phosphorylated NF-κB and STAT3 expression from B-cells, T-lymphocytes and monocytes/macrophages**. Summary of flow cytometry analysis (n = 7) of percentage of phosphorylated NF-κB (A) and phosphorylated STAT3 (B) activation (versus untreated) from B-cells, T-lymphocytes and monocytes/macrophages. PBMCs were stimulated for the appropriate time and concentration of sCD40L (A) and IL10 (B) (as identified previously). The graphs represent the difference in percentage of phosphorylated nuclear factor between stimulated and untreated cells. Statistical significance (wilcoxon paired test; p < 0.05) was represented by an asterisk (*). Data represented the mean (± SD) of seven experiments.

Induction of pNF-κB translocation following stimulation with sCD40L used as predetermined, optimal concentrations, was observed in B-cells and monocytes/macrophages – but, interestingly – not in T-lymphocytes; phosphorylation of NF-κB was significantly (p < 0.05) increased vs baseline levels, by a magnitude of 6.31 ± 1.64% in B-cells and of 11.75 ± 4.73% in monocytes/macrophages (Figure 3A).

In parallel, Figure 3B represents the percentage of phosphorylated STAT3 of the various PBMC cell types in response of optimal IL10 stimulation of unseparated cultured cells. There is a significant (p < 0.05) increase (vs baseline levels) of nuclear STAT3 translocation in each cell type, by a magnitude of 25.85 ± 6.97% in B-cells, of 7.99 ± 4.67% in T-lymphocytes and of 11.00 ± 4.72% in monocytes/macrophages.

## Discussion

A recent review [30] states that the development of phospho-specific intracellular detection necessitates development of reagents and adapted protocols. Nuclear translocation of transcription factors can be usually measured by immunoblotting of isolated nuclei by electrophoretic mobility shift assay (EMSA), or more recently in flow cytometry on purified cells [4,27]. Aubry and coworkers described in 2002 an original technique to study the NF-κB pathway by multichannel flow cytometry after permeabilisation of DCs stimulated with LPS [27]. Next, our group contributed in 2003 an original approach to study the phosphorylated transcription factor NF-κB in purified B-cells populations originating from blood or lymphoid tissues, with the need to purify the target cells to homogeneity. The main problem of this technique is the loss of information because of not studying cellular events under physiological conditions. Now, we understand the influence of the culture environment on any cell and the importance of studying cells within their context. So scientists need more and more techniques which study specific cells in their environment. In this report, we have developed and validated a flow cytometry to study transcription factors for various cell subpopulations within one cell type or a tissue suspension (we chose PBMCs), because there is a need to study rapidly, commonly and reproducibly such parameters (nuclear factor phosphorylation/translocation) in blood samples representing steady state or pathological conditions. Phosphorylation of proteins in signal transduction is traditionally studied by means of Western Blot Analysis and/or ELISA, which carry valuable information regarding homogeneous or purified cell populations. The present study proposes an original method to study phosphorylation of proteins in complex, heterogeneous, cell populations such as unseparated PBMCs. This method could be further validated to be applied to whole blood samples obtained from patients presenting with inflammatory diseases, for example, where there is a need to study cellular events [31]. Indeed, the NF-κB pathway plays a pivotal role in immune and inflammatory responses and has attracted much interest as a novel target for the treatment of inflammatory states. Meanwhile, the major interest resides in the identification of cell subsets displaying altered nuclear factor phosphorylation/translocation, thus to be clearly identified by surface membrane characteristics.

Multiparameter flow cytometry offers the valuable advantage of characterising, almost without doubt, certain cell surface markers to identify subpopulations in response of a stimulus. In this study, we used 3 labelling conditions: one for each cell type, one for DNA gating and one for the intracellular factor of interest. In this manner, we marked only one cellular type and one intracellular factor per tube. However, by using a flow cytometer with more excitation lasers, it could be possible to mark DNA, all types of cells and the 2 intracellular factors in the same tube. Here we confirm that intracellular pathways of NF-κB and STAT3 can be rather easily monitored by Western Blot and ELISA (we thus took advantage of these reproducible techniques to set up optimal conditions for the stimulation of PBMCs with external signals leading to the use of either NF-κB or STAT3, that are for instance IL1β and sCD40L (NF-κB) and IL10 (STAT3), according to a general consensus [23]. The development of a multiparameter flow cytometry technique permits us to decipher which of the stimulated cell population studied responded to treatment. We evidenced that IL1β and sCD40L should be used at 50 ng/mL for 30 min and IL10 at 100 ng/mL for 30 min. From the above experiments – principally aiming at presenting a convenient technique for studying and foremost tracing cellular translocation of – for the time being NF-κB and STAT (but – hopefully – additional pathways such as other STATs and also SMAD) – we could provide evidence that unseparated monocytes/macrophages responded strongly to sCD40L and B-cells to IL10. This result is not surprising because the literature showed the activation of PBMCs with these cytokines [32]. This study has no real impact on the results but on the technique, itself. The obtained results proved this method is valuable to study activation of cells.

In flow cytometry, only 4 hours are necessary to prepare the samples and roughly 1 hour to acquire and analyse the data. It is the most feasible technique, however, the use of the cytometer and analyse software require training. Flow cytometry is not the most sensitive technique but the sensitivity is largely acceptable (1 μg/tube). Relating to the reproducibility, we observe a difference of the basal rate of activation depending on the individual samples. Regulated degradation of specific proteins is part of the intracellular biochemical changes that contribute to the regulation of signal transduction pathways; NF-κB activation thus, requires the rapid degradation of IκBα, the inhibitor of NF-κB. Before activation, NF-κB is sequestered in the cytoplasm by forming a complex with IκBα, the nuclear translocation signal of NF-κB being masked by the inhibitor. NF-κB can be activated by a number of stimuli, which trigger the phosphorylation of IκBα and lead to the rapid dissociation of NF-κB from IκBα. Once released from the IκBα complex, NF-κB immediately translocates from the cytoplasm to the nucleus, where it mediates the transcriptional activation of genes. At this point of phosphoprotein translocation, it is difficult to see the effective translocation by flow cytometry. 7-Amino-Actinomycin D (7-AAD) is a convenient, ready-to-use nucleic acid dye that can be used in flow cytometric assays for exclusion of nonviable cells, or to gate nuclei and label nucleic acids, in replacement of propidium iodide (PI) in flow cytometric assays [4]. The advantage of 7-AAD over PI is its ability to be used in conjunction with phycoerythrin (PE) – and fluorescein isothiocyanate (FITC)-labelled monoclonal antibodies in 2-color analysis, with minimal spectral overlap. Therefore, the relationship of basal degradation to induced degradation remains uncertain [33]. Moreover, NF-κB regulation of IκBα transcription represents a delayed negative feedback loop that drives oscillations in NF-κB translocation. Transcription of target genes depended on oscillation persistence, involving cycles of RelA phosphorylation and dephosphorylation [29]. The oscillations in NF-κB signalling can explain the background levels of pNF-κB/pSTAT3 positive cells in unstimulated PBMCs. Nevertheless, a similar difference between stimulated and non stimulated cells is always detected.

This paper describes a novel flow cytometry method which allows the detection and quantification of intranuclear levels of the transcription factors NF-κB and STAT3 in distinct PBMCs subpopulations; this technique enables the identification of CD3, CD14 and/or CD19 subsets – within unseparated PBMCs (and, maybe in the future, whole blood cells) – which actually phosphorylate intranuclear proteins upon appropriate stimulation. These strategies will enable the development of simple though robust blood methods to identify normal signalling pathways as well as impairments in phosphorylating proteins in association with various human disease states (including acute and chronic inflammatory conditions), or with other chronic diseases characterized by an alteration of the cytokine homeostatic milieu, as seen in profound allergies or in cancer states [34].

In this way, our data stress the differences that are detectable between lymphocytes and monocytes regarding NF-κB or STAT3 phosphorylation upon relevant stimulation; an explanation could be because monocytes are associated with innate immune responses faster than, for example, T or B-cells, an issue which would deserve closer examination.

In summary, to determine the optimal conditions of activation, ELISA is the most practical technique to study at the same time a large quantity of samples. Once the optimal conditions have been identified, the Western Blot and the flow cytometry technique become the most adequate techniques, although the flow cytometry remains the most rapid method.

## Conclusion

Our technology to study transcription factors, such as pNF-κB and/or pSTAT3, could be applicable to infrequent cell subpopulations such as stem cell progenitors or other cells with various stages of maturation/differentiation [35]. Moreover, this original technology can be used to detect transcription factor translocation; for example to screen a new molecule that induces a cell response.

This flow cytometry technique is very interesting because it can be applicable to different types of cellular relationship studies, in particular in blood transfusion, immunology and oncology. Indeed, this method could quantify the activation of immune cells of a transfused person or of a person who received platelets in order to better limit the response. Concerning oncology, the NF-κB and STAT pathways play important roles in tumorigenesis [36]. Thus, these intracellular pathways appear very interesting to study in an oncological context. So, this new flow cytometry technique appears very attractive for signal study in PBMCs or tissue conditions.

## Methods

### Cell isolation

Human blood was obtained from healthy donors at the Auvergne-Loire Regional Blood Bank. Peripheral Blood Mononuclear Cells (PBMCs) were prepared from Buffy-Coats as described previously [28]. PBMCs were isolated by gradient density centrifugation using histopaque-1077 (Sigma-Aldrich, Saint Quentin Fallavier, France).

### Cell stimulation

PBMCs were cultured at 20 × 10^6^/mL in IMDM (Iscove Modified Dulbecco's Medium) (Sigma-Aldrich) supplemented exactly as described previously [28]. PBMCs were stimulated in the presence or absence of IL1β (AbCys, Paris, France), purified – soluble – trimeric human CD40L molecules (sCD40L) [37] (Alexis-Coger, Paris, France) or IL10 (R&D System, Lille, France) at varying concentrations: IL1β (0 to 200 ng/mL), sCD40L (0 to 200 ng/mL) and IL10 (from 0 to 200 ng/mL) and for various time periods (0, 5, 15, 30 min). Stimulated PBMCs were incubated in a humidified atmosphere at 37°C with 5% CO_2_.

### Nuclear and cytoplasmic extraction

After incubation, PBMCs were washed with PBS containing 10% endotoxin-free FBS (Cambrex, Verviers, Belgium). Washed cells were then treated with a nuclear extract kit (Active Motif, Rixenart, Belgium) according to the manufacturer's instructions. A cytoplasmic extract and a nuclear extract for each culture condition were obtained and conserved at -80°C until further assays.

### Transcription factor ELISA

pNF-κB (phosphorylated NF-κB) p65 subunit and pSTAT3 (phosphorylated STAT3) levels in nuclear extracts from stimulated vs non-stimulated PBMCs were determined by ELISA for each PBMCs culture condition. pNF-κB (assay sensitivity = 0.5 μg/well) and pSTAT3 (assay sensitivity = 0.6 μg/well) was detected using a transcription factor ELISA kit (Active Motif). Briefly, 2.5 μg of each nuclear extract was incubated in 96-well plates containing a consensus (5'-GGGACTTTCC-3') binding site for the p65 subunit of pNF-κB. pNF-κB binding to the target oligonucleotide was detected by incubation with a primary antibody specific for the activated form of p65, and then visualized by incubation with anti-IgG horseradish peroxidase conjugate at optimal concentrations and a developing solution as described by the manufacturer, and quantified at 450 nm with a reference wavelength of 655 nm (Multiskan EX, Labsystem, VWR International, Strasbourg, France). The principle is the same for pSTAT3 with a specific consensus binding site (5'-TTCCCGGAA-3') and transcription factor ELISA to detect STAT3 phosphorylation was performed as described by the manufacturer.

### Detection of nuclear factors by Western Blot

Twenty-five micrograms of nuclear extract per well were separated by 10% acrylamide gel (Sigma-Aldrich) and transferred to a 0.45 μm nitrocellulose membrane (Amersham Pharmacia, Orsay, France) by electroblotting using transfer buffer supplemented with 20% methanol (Sigma-Aldrich). Blots were blocked overnight at 4°C in PBS/0.1% Tween 20/1% BSA (I.D. Bio, Limoges, France) and incubated with a primary antibody to pNF-κB (0.4 μg/mL) (Santa Cruz Biotechnology, Montrouge, France) or to pSTAT3 (0.4 μg/mL) (Santa Cruz Biotechnology), for 90 min, at room temperature. Thereafter, the blots were washed three times for 10 min with blocking buffer, then incubated for other 90 min with the secondary horseradish peroxidase-linked goat anti-rabbit antibody diluted at 1:5000 (Santa Cruz Biotechnology) as predetermined. Then, blots were incubated with a chemiluminescent substrate according to manufacturer's instructions (ECL; Amersham Pharmacia) and finally exposed to radiographic film (Sigma-Aldrich).

### Detection of nuclear translocation by flow cytometry

After stimulation, PBMCs were washed with PBS containing 10% endotoxin-free FBS (BioWest) and fixed with PBS/PAF 1% (Sigma-Aldrich) at room temperature for 20 min. (the same result was obtained at 37°C). PBMCs sub-populations were labeled by FITC monoclonal antibodies (5 μg/10^6 ^cells): CD19-FITC (BD Biosciences, Le Pont de Claix, France) for B-cells, CD14-FITC (BD Biosciences) for monocytes/macrophages and CD3-FITC (BD Biosciences) for T-lymphocytes, in separated tubes at 4°C for 45 min. After two washes, PBMCs were permeabilized by 0.03% saponin (Sigma-Aldrich) at room temperature for 15 min. After two washes in PBS/FBS, PBMCs were stained with PE labeled-anti-pNF-κB antibody (2 μg/10^6 ^cells) (Santa Cruz Biotechnology) or PE labeled-anti-pSTAT3 antibody (2 μg/10^6 ^cells) (Santa Cruz Biotechnology) for 60 min at room temperature. Then, PBMCs DNA was counterstained with 7AAD (0.25 μg/10^6 ^cells) (BD Biosciences) at room temperature for 10 min. Finally, after washing in PBS-FBS, PBMCs sub-populations were analyzed by flow cytometry with a FACSCalibur (BD Biosciences). The software used was CellQuest ProTM (BD Biosciences). A total of 10^4 ^events was recorded for each sample. Experiments were reproduced in order to set up conditions allowing fine reproducibility of the technique with limited inter-individual variation for a given culture condition.

### Statistical analysis

Inter-experiment comparisons in stimulated vs unstimulated PBMCs were analyzed by means of the Wilcoxon paired test (StatviewTM, SAS Inst., Cary, NC). Differences were considered statistically significant with values of p < 0.05.

## Authors' contributions

SL designed, performed the research, analyzed the data and wrote the first draft. HHC designed, performed the research and analyzed the data. PC performed the research. CG performed the research. OG designed the research, analyzed the data, and wrote the first and second draft. FC designed, performed the research, analyzed the data and wrote the first and second draft. All authors read and approved the final manuscript.
